# The prognostic value of miR-126 expression in non-small-cell lung cancer: a meta-analysis

**DOI:** 10.1186/s12935-017-0440-8

**Published:** 2017-07-14

**Authors:** Wen Zheng, Ya Zhou, Jia Lu, Hualin Xu, Liangyu Lei, Chao Chen, Juanjuan Zhao, Lin Xu

**Affiliations:** 1 Department of Immunology, Zunyi Medical College, Guizhou, 563000 People’s Republic of China; 2 Department of Medical Physics, Zunyi Medical College, Guizhou, 563000 China

**Keywords:** miR-21, NSCLC, Prognosis, Meta-analysis

## Abstract

**Objective:**

Non-small cell lung cancer (NSCLC) is a leading cause of cancer-related death. Growing evidence from recent studies have shown indicated that microRNA-126 (miR-126) played an important role in the progression of NSCLC. However, the potential value of miR-126 expression in prognosis of NSCLC remains to be fully elucidated. Here, we carried out a meta-analysis to assess the potential prognostic value of miR-126 for NSCLC.

**Methods:**

PubMed, Embase, the Cochrane library, Web of Science, CNKI and WanFang database, as well as the reference of included studies, were searched to recognize pertinent studies until April 30, 2017. New castle-Ottawa scale was used to evaluate the quality of studies. Pooled hazard ratio (HR) with 95% confidence interval (CI) for overall survival (OS) was extracted by using a fixed-effects or a random-effects model on the basis of heterogeneity. Publication bias was evaluated by using Begg’s tests.

**Results:**

We identified four eligible trials involving 666 non-small-cell lung cancer patients in this meta-analysis. The results indicated that a high level of miR-126 played a favorable role in the overall survival (HR 0.73, 95% CI 0.61–0.86, fixed-effects model). There was no bias existed in this study.

**Conclusions:**

Our study showed that high expression level of miR-126 was a promising positive factor for OS for non-small cell lung cancer patients, and miR-126 might be a potential target for non-small-cell lung cancer therapy in the future.

## Introduction

Lung cancer is the leading cause of cancer-related deaths in the world, and nearly 1 million new cases are expected annually by 2025, and non-small-cell lung cancer (NSCLC) accounts for 80% of cases [1–3], including major subtypes such as lung adenocarcinoma (ADC), squamous cell carcinoma (SCC), and large cell carcinoma (LCC) [4]. Although novel therapies have been developed, 5-year survival rate was still less than 15% due to its late diagnosis and poor outcome [5]. Therefore, further studies on prognostic assessment of these patients are essential for treatment stratification and ideal outcome of clinical lung cancer patients.

MicroRNAs (miRNAs) are small non-coding RNAs that negatively regulate gene expression through complementarity between the miRNA seed sequence and the target mRNA 3′untranslated region (UTR) [6–8]. Up to data, it was estimated that miRNAs may potentially regulate up to 30% of all human protein-coding genes [9], Recently, a large body of evidence showed that miRNAs exerted pivotal effects in the development and progression of human malignancies, including NSCLC [10]. As an important member of the miRNA family, microRNA-126 (miR-126), located within the seventh intron of epidermal growth factor-like protein 7 gene [11], was participated in a wide range of biological functions. Such as, in our previous study, we found that miR-126 could regulate the induction and function of CD4^+^Foxp3^+^ regulatory T cells through PI3K/AKT pathway [12]. Notably, MiR-126 also was documented played a vital role in the progression of NSCLC, For example, the expression level of miR-126 was significantly downregulated in NSCLC [13–22]. Moreover, miR-126 could control the biological characters of NSCLC through different mechanisms. For instance, miR-126 could inhibit the proliferation of NSCLC through EGFL7 [23]. The upregulation of miR-126 in NSCLC A549 cells could reduce the expression of the target gene PIK3R2 and influence the PTEN/PI3K/AKT signaling pathway, suppressing the proliferation, migration, and invasive abilities of A549 cells [24]. In addition, decreased miR-126 expression could enhance the adhesion, migration and invasion of NSCLC cells through increased Crk protein [16, 25]. These results suggested that miR-126 may be function as an important regulatory gene in the development of NSCLC.

Furthermore, it has been reported that miRNA-126 has a potential prognostic role for predict outcome of non-small-cell lung cancer. However, there has been no systematic review of these literatures. Therefore, we firstly performed a meta-analysis of the data available from articles published in this field to evaluate the role of miR-126 expression as prognostic biomarker in non-small-cell lung cancer.

## Materials and methods

### Search strategy

Several relevant literature databases (PubMed, Embase, The Cochrane Library and Web of Science) and Chinese databases (CNKI and Wan Fang database) were searched for studies that estimated the diagnostic value of miR-126 in non-small-cell lung cancer. The studies were selected by using the following keywords in various combinations: ‘microRNA-126’, ‘miRNA-126’, ‘microRNA126’, ‘miR-126’, ‘miRNA126’, ‘miR-126-3p’, ‘carcinoma’, ‘non-small-cell lung’, ‘lung neoplasms’, and ‘non-small cell lung cancer’. The database was searched for the last time on April 30, 2017. The comprehensive database search was carried out independently by two authors.

### Selection criteria

We used references manager software EndNote to check out duplications. The studies were selected and the data were extracted independently by two authors. And according to the follow inclusive criteria to choose studies: (1) it studied miR-126 in non-small-cell lung cancer; (2) it must be human samples; (3) it studied the association between miR-126 and survival outcome.

The exclusion criteria were the following: (1) studies related to the associations between miR-126 expression and prognosis that did not include survival analysis; (2) investigation of a set of miRNAs but not miR-126 alone. And (3) letters, case reports, reviews, conference abstracts, and animal or laboratory studies. (4) or lack of important information such as hazard ratio (HR), 95% CI and *P* value. (5) hazard ratio (HR) underwent subgroup analysis of pathological types of NSCLC.

### Quality assessment

Newcastle–Ottawa scale was used to assess the methodological quality of studies incorporated in this meta-analysis [26], This standard assessed 3 sections (selection, comparability, exposure) and eight items. In the selection and exposure categories, a quality research item received 1 star, and a comparable category could receive at most two stars. The quality assessment values ranged from 0 to 9 stars. Generally, the study which scored at least five points was considered to be included in meta-analysis. The lowest score was 0 and the highest was 9. Studies with a score <5 in the present study were not included in the final analysis.

### Data extraction

The data elements of this review including the following: (1) first author’s name, publication year, tumor grade, total number of Sample, miR-126 assay and cut-off values, (2) HR with 95% CI, outcomes and median follow-up (months). If the data were not provided visually and were only provided as Kaplan–Meier curves, the data were extracted from the graphical survival plots, and estimations of the HRs were then performed using a previously described method.

### Statistical analyses

All data were pooled using STATA, version 12.0 (Stata Corp., College Station, TX). HR with 95% CI was used to combine the pooled data. We used the Chi square based Q-test to test the statistical heterogeneity of studies, then identified the absence of heterogeneity across studies, after used fixed-effects model (the Mantel–Haenszel method), Heterogeneity was defined as *P* < 0.10 or I^2^ > 50%. Finally, we used the methods of Begg plots test to analysis publication bias (*P* < 0.05 was considered representative of statistically significant publication bias).

## Results

### Literature research and characteristic of studies

**Fig. 1:**
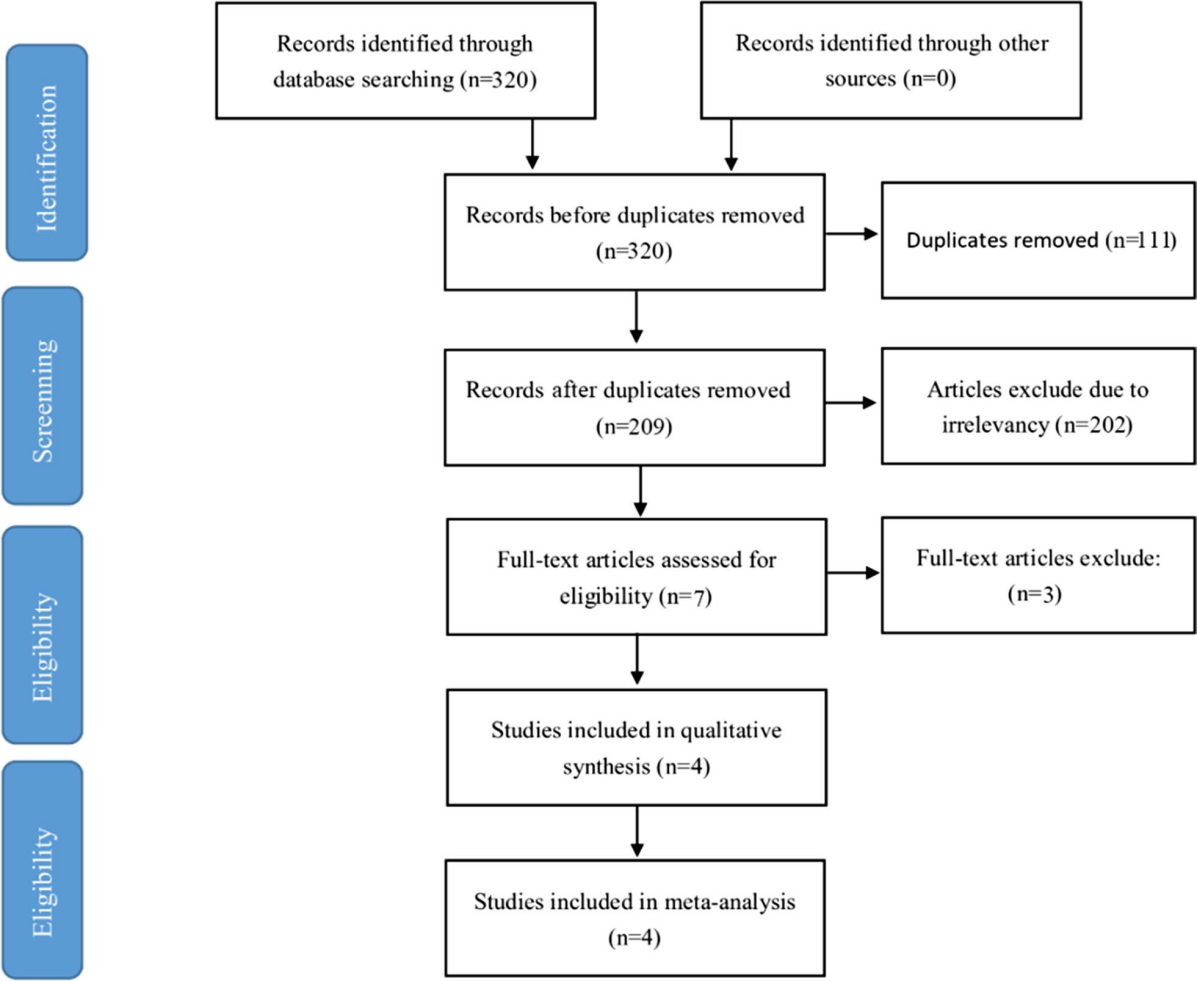
Flow diagram of the studies identification and selection

A total of 320 studies were identified from the databases. 111 were duplicates, and the remaining 209 were further screened. Based on readings of the article titles and abstracts, as well as according to the inclusion and exclusion criteria, seven studies were selected for further investigation (as shown in Fig. 1). After reading the full texts of the remaining, 2 articles were excluded because they were recorded disease-free survival (DFS) and disease-specific survival (DSS) [27, 28], 1 articles were excluded because hazard ratio (HR) with 95% confidence interval (CI) for overall survival (OS) undergo subgroup analysis of pathological types of NSCLC [29]. Then, A total of 4 articles were eventually included [30–33]. These four studies included a total of 666 patients. The method of miR-126 detection was all quantitative real-time polymerase chain reaction. MiR-126 expression level were measured in tumor tissue. The clinicopathological characteristics of the eligible studies are summarized in Table 1.

**Table 1:** Characteristics of the studies included in this meta-analysis

Study	Year	Country	Sample number	Specimen	Stage	miR-126 assay	Cut-off value	Hazard ratio	Endpoint	Follow-up (months)
Chen et al. [30]	2015	China	103	Tissue	I–III	qRT-PCR	Median	DE	OS	NR
Kim et al. [31]	2014	South Korea	72	Tissue	I–IV	qRT-PCR	Median	Report	OS	31
Li et al. [32]	2014	China	49	Tissue	NR	qRT-PCR	Median	DE	OS	39
Yang et al. [33]	2012	China	442	Tissue	I–IV	qRT-PCR	Median	Report	OS	24.39–29.28

*NR* not reported, *qRT-PCR* quantitative real-time polymerase chain reaction, *OS* overall survival, *DE* data extrapolated

### Quality assessment

The details of quality assessment based on the NOS are shown in Table 2. The last column in each row listed the total score of each study. Newcastle–Ottawa scale revealed that the study quality varied from 7 to 8 (Table 2). The quality of included studies was good and fair.

**Table 2:** Quality assessment based on the Newcastle–Ottawa scale

Study	Selection	Comparability	Exposure	Total
Chen et al. [30]	4	1	1	6
Kim et al. [31]	4	1	2	7
Li et al. [32]	4	1	2	7
Yang et al. [33]	4	2	2	8

*Selection* representativeness of studies (maximum score of 4), *Comparability* comparability of studies based on the design or analysis (maximum score of 2), *Exposure* assessment of outcome and follow-up (maximum score of 3)

### Meta-analysis

**Fig. 2:**
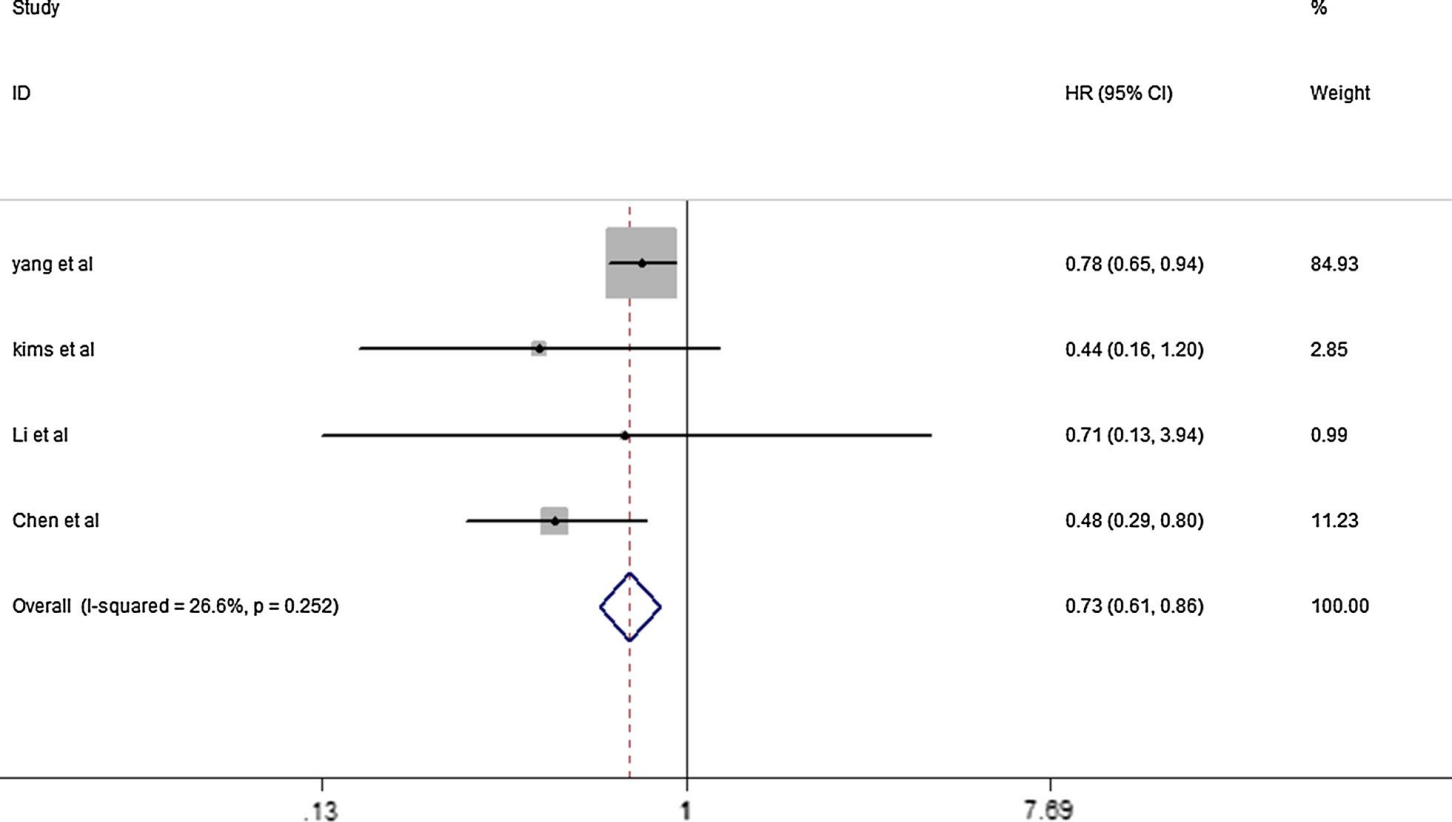
Forest plots of studies evaluating the pooled HR of elevated miR-126 level for overall survival (OS)

For evaluating the association between miR-126 expression and OS for NSCLC, Forest plots of the individual HR estimates and the results of the meta analysis are presented in Fig. 2, No heterogeneity was detected between the studies, Therefore, a fix effects model was applied to calculate a pooled HR and its 95% CI (I^2^ = 26.6%, *P* = 0.252). These results showed that a higher expression level of miR-126 has significantly positive prognostic role in NSCLC and the pooled HR was 0.73 (95% CI 0.61–0.86).

### Publication bias

**Fig. 3:**
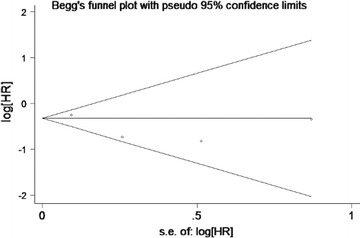
Begg’s funnel plots of publication bias test for overall survival (OS)

We used funnel plots and Begg’s tests to evaluate the publication bias of included studies. *P* < 0.05 indicated the presence of publication bias. The funnel plot of OS analysis was revealed in Fig. 3, and the *P* value of Begg’s regression intercept was 1.000, indicating that no evidence of significant publication bias was found in this meta-analysis.

## Discussion

MiR-126 has played a wide variety of function in non-small cell lung cancer. Such as, it was found at the cellular level, the upregulation of miR-126 in NSCLC A549 cells could reduce the expression of the target gene PIK3R2 and influence the PTEN/PI3K/AKT signaling pathway, suppressing the proliferation, migration, and invasive abilities of A549 cells [34]. Restoration of hsa-miR-126 more obviously inhibited cell growth, and the suppressive effect was more significant in H460 xenografts, which resulted from the inhibition of the activation of Akt and ERK [35]. Moreover, miR-126 was involved in regulating the response of NSCLC cells to cancer therapy. For example, miR-126 could promote non-small cell lung cancer cells apoptosis induced by irradiation through the PI3K-Akt pathway [36]. Enhanced expression of miR-126 could increase the sensitivity of NSCLC cells to anticancer agents through negative regulation of a VEGF/PI3K/Akt/MRP1 signaling pathway [37]. Furthermore, MiR-126 might be contributed to enhanced cytotoxicity induced by Gefitinib in lung cancer cells [35]. In addition, Matrine, as an active component of traditional Chinese medicine, could effectively induce cell cycle arrest and apoptosis, and recovered the expression of miR126 in NSCLC A549 cells [38]. These studies indicated that miR-126 played a key role in the biology of NSCLC cells, indicating it was a promising target for gene therapy against NSCLC patients.

Interestingly, recent evidence further showed that the expression level of miR-126 also changed in clinical NSCLC patients [39]. Such as, lower level of miR-126 was associated with poor pathological stage [14], large tumor diameter and lymph node metastasis [30, 40]. Recently, Shang et al. [41] reported that the specificity and sensitivity of serum miR-126 level in predicting NSCLC development were 82.68 and 96.40%, respectively. Meanwhile, the specificity and sensitivity of serum miR-126 level in predicting NSCLC metastasis were 84.00 and 62.30%. This study indicated that serum miR-126 level may be used as the predicative biomarkers for NSCLC development and metastasis. Furthermore, there was statistical difference on the serum level of hsa-miR-126 between in the Stage IV NSCLC patients and in the health control [42]. And, miR-126 was independently associated with a dismal prognosis (HR 4.1, 95% CI 2.0–8.4, *P* < 0.001) in lymph node-positive patients [28]. Further studies showed that miR-126 also played an important role in the diagnosis of non-small-cell lung cancer patients [43, 44]. For example, Zhu et al. revealed that the serum miR-126, miR-182, miR-183 and miR-21 levels could serve as a diagnostic biomarker for NSCLC early detection, with a high sensitivity and specificity (sensitivity, 81.3%; specificity, 100.0%; and accuracy, 90.8%) [45]. Expression levels of miR-126, -296, -145, -199a, -191, -223, -24, -152, -320, and let-7 in the plasma of NSCLC including stage-I patients were significantly higher than these in controls. The combination of these microRNAs yielded 87% sensitivity and 90% specificity (AUC = 0.934) in discriminating NSCLC patients from controls [46]. Similarly, an eleven-plasma miRNA panel (including miR-126) that could distinguish NSCLC patients from healthy subjects (AUC = 0.879) [27]. Song et al. also reported that combination of three miRNAs miR-126, miR-205 and miR-182 could also predict NSCLC at the accuracy of 84.49%, sensitivity of 91.40% and specificity of 77.14%, respectively [47]. Interestingly, exosomes miR-126 could also be reported as a marker of diagnosis of NSCLC patient. For example, Exosomes miR-126 and let-7a were present in significantly higher levels in the BAL fluid of lung adenocarcinoma patients, and they could serve as diagnostic biomarkers in early stage lung adenocarcinoma [48]. Combining these data showed that miR-126 was a potential biomarker for the diagnosis of NSCLC.

Notably, MiR-126 has also been reported was a promising index in the prognosis of NSCLC patients. For Example, the levels of miR-126 were significantly associated with disease free survival (DFS) rate [32, 49, 50]. And, low plasma level of miR-126-3p were significantly associated with poor DFS in lung adenocarcinoma patients(HR = 0.497,95% CI = 0.191–1.295). Jusufović et al. discovered that lower miR-126 expression was a negative prognostic factor for both progression free survival (HR 0.10, 95% CI 0.04–0.21) in NSCLC patients, which may be attributed to elevated tumor angiogenesis [29]. In line with these findings, in present study, our analysis showed that elevated miR-126 expression alone also could be positively related to the overall survival (OS) in patients with NSCLC, indicating an important value of miR-126 expression alone in the prognosis of NSCLC patients. Interestingly, Lønvik et al. also reported that Drosha/miR-126 coexpression had a significant negative impact on the disease-specific survival (DSS) rate (*P* < 0.001) [51]. Donnem et al. also demonstrated that coexpression of VEGF-A and miR-126 had an independent prognostic impact (*P* = 0.017). For NSCLC patients with high VEGF-A/high miR-126 expression, HR was 2.5 (95% CI = 1.4–4.3) on the disease-specific survival (DSS) compared with patients who had low VEGF-A/low miR-126 expression [28]. Furthermore, NSCLC patients with low expression of miR-126 or both miRNAs combined (let-7b and miR-126) were highly associated with Progression-free survival (PFS), HR = 0.22 (95% CI = 0.07–0.74) or HR = 0.09 (0.01–0.65) [29]. However, the possible difference and significance between the expression of miR-126 alone and the coexpression of miR-126 and other factors on the prognosis of NSCLC patients still remained to be elucidated in future. Finally, it should be noted that Jusufovic et al. reported overall survival (OS) in patients with NSCLC (HR = 0.33 95% CI = 0.09–1.21) [29], which did not be included in this study. Because we found that HR for overall survival (OS) were obtained from subgroup analysis of pathological types of NSCLC, which was different from the other 4 literatures included in current study. Interestingly, we also noticed that the patients of this article were from Serbia, however, all of the patients in our study were from Asia, which could lead to increased heterogeneity (I^2^ = 78.8%, *P* = 0.001). Therefore, we presumed that difference of racial and pathological types might be potential cofactors for the prognosis of miR-126 expression in NSCLC, which was much valuable to be validated in successive research work.

Although, miR-126 has been widely investigated, however, there are still some limitations to be considered. Firstly, only four studies were eligible for pooled analysis, which might affect the results of the present study. Secondly, the expression of miR-126 was detected in tumor tissue samples but not in serum or plasma. However, circulating prognostic markers were found to be more valuable than tissue markers in cancer patients. Therefore, the conclusions from our study should be tempered. Thus, further large studies taking into account the effects of gender, age and ethnicity, just as mentioned above, are warranted to obtain a more robust assessment of the association between miR-126 and the prognosis of NSCLC. In conclusion, our results supported a value prognostic role for miR-126 in NSCLC, which might be ultimately useful for the understanding on the role of miR-126 in occurrence of NSCLC and development of effectively therapeutic agents of NSCLC patients.

## Conclusion

Our study showed that high expression level of miR-126 was a promising positive factor for OS of non-small cell lung cancer patients, which indicated that miR-126 might be a potential target for non-small-cell lung cancer therapy and ultimately benefit the outcome of clinical therapy in the future.

## Abbreviations

NSCLC: non-small cell lung cancer
miR-126: microRNAs-126
OS: overall survival
PFS: progression-free survival
DSS: disease-specific survival
CI: confidence interval
